# Study of the volatilization rules of volatile oil and the sustained-release effect of volatile oil solidified by porous starch

**DOI:** 10.1038/s41598-022-11692-w

**Published:** 2022-05-17

**Authors:** Guilin Ren, Gang Ke, Rui Huang, Qingrong Pu, Jian Zhao, Qin Zheng, Ming Yang

**Affiliations:** 1 The Affiliated Traditional Chinese Medicine Hospital of Southwest Medical University, Luzhou, 646000 China; 2 Luzhou People’s Hospital, Luzhou, 646100 China; 3 Key Laboratory of Modern Preparation of Traditional Chinese Medicine, Ministry of Education, State Key Laboratory of Innovation Drug and Efficient Energy-Saving Pharmaceutical Equipment, Jiangxi University of Traditional Chinese Medicine, Nanchang, 330004 China

**Keywords:** Chemistry, Materials science

## Abstract

Volatile oil from traditional Chinese medicine has various biological activities and has pharmacological activities in the central nervous system, digestive system, cardiovascular system, respiratory system, etc. These oils are widely used in clinical practice. However, the development of their clinical applications is restricted due to the disadvantages of volatile oils, such as high stimulation, high volatility and poor stability. To improve the stability of a volatile oil in the preparation process, its volatilization and stable release must be controlled. In this paper, porous starch was used as a solid carrier material, and liquid volatile oil was solidified by physical adsorption. GC–MS was used to determine the chemical constituents of the volatile oil, solidified powder and tablets, and the volatilization rules of 34 chemical constituents were analysed statistically. The solidified volatile oil/porous starch powder was characterized by XRD, TGA and DSC, and the VOCs of the volatile oil before and after solidification were analysed by portable GC–MS. Finally, the stable release of the volatile oil could be optimized by changing the porous starch ratio in the formulation. Volatilization was shown to be closely related to the peak retention time and chemical composition, which was consistent with the theory of flavour. The physical properties and chemical composition of the volatile oil did not change after curing, indicating that the adsorption of the volatile oil by porous starch was physical adsorption. In this paper, the porous starch-solidified volatile oil had a slow-release effect, and the production process is simple, easy to operate, and has high application value.

## Introduction

The volatile oils (VOs) of traditional Chinese medicine have various biological activities, such as cough relief, asthma relief, anti-bacterial, pain relief, spasm relief, anti-cancer and stomach strengthening effects^1–5^. VOs have has pharmacological activities in the central nervous system, digestive system, cardiovascular system and respiratory system and have been widely used in clinical applications^6–10^. However, VOs can evaporate at room temperature and are sensitive to air, light and heat, which limits their application. Therefore, it is particularly important to improve the stability of VOs.

At present, the main way to improve the stability of VOs is inclusion technology, of which β-cyclodextrin is widely used, but the technology has the problems of a low encapsulation rate and use of a large amount of excipients^11–14^. In recent years, modern preparation technologies, such as microencapsulation, microspindling, liposomes and nanoemulsions, have also been used to stabilize VOs of traditional Chinese medicine, but the application of these technologies has the disadvantages of organic solvent residues, a low drug load, poor stability and unknown toxicity and side effects, which therefore demand extensive preparation processes that are not conducive to large-scale industrial production^15–17^.

To improve the stability of VOs in the preparation process, their volatilization and stable release must be optimized. In this paper, the volatile oil of dried tangerine peel and immature tangerine peel was extracted by steam distillation. The VO was taken as the research object, and porous starch (PS) was selected as the carrier material to absorb the VO. Through physical adsorption, the liquid materials were solidified with a simple stirring method in which the carrier and VO could be evenly mixed, improving the stability of the oil. Moreover, the powder could be prepared in a variety of dosage forms, as the preparation technology is simple, convenient, and low cost. Thus, the developed method has good application prospects.

**Figure 1:**
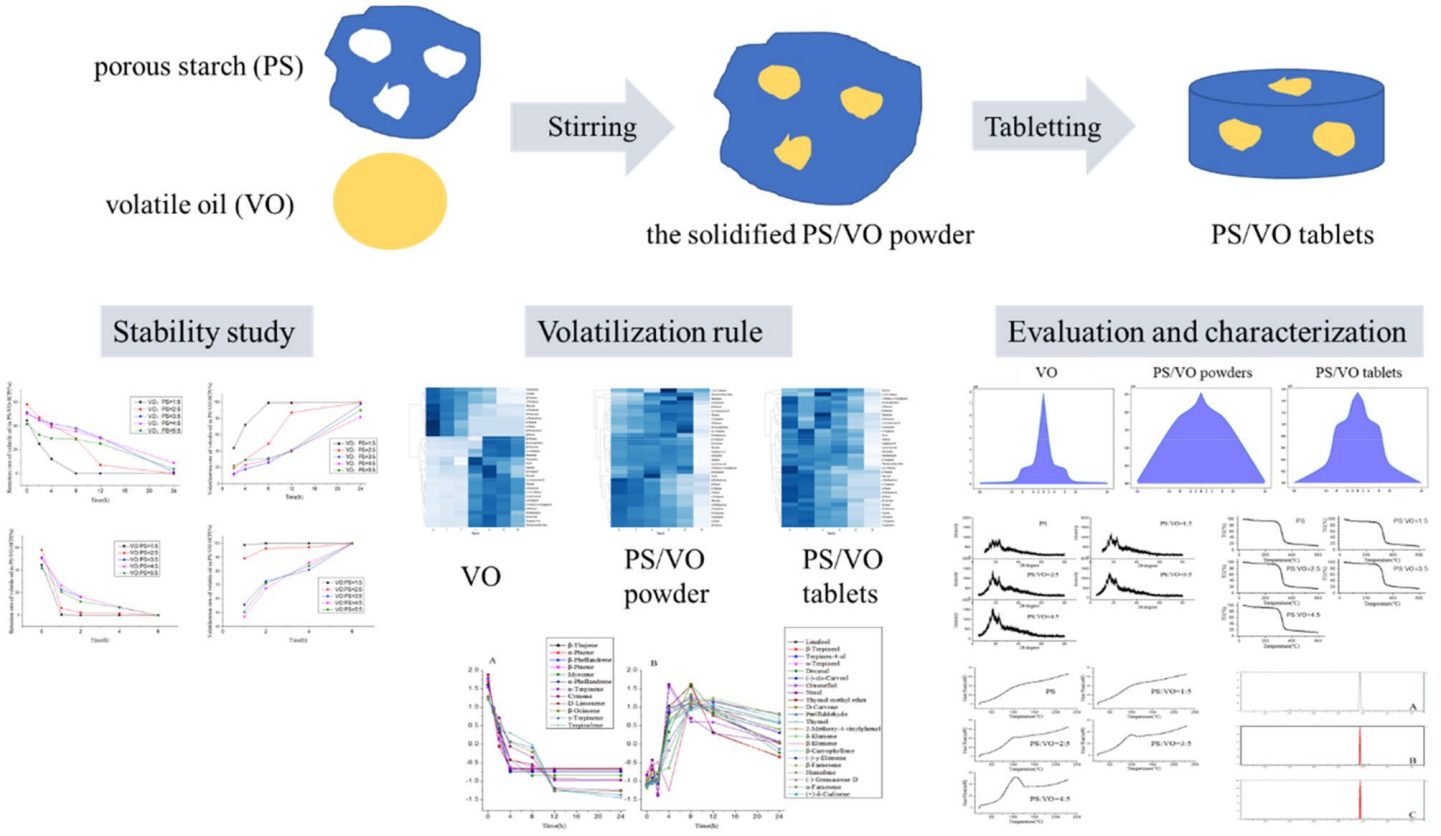
The experimental flow chart.

In this study, GC–MS was used to determine the chemical composition of the VO and solidified powder, and the volatilization rules of the VO constituents were studied. X-ray diffraction (XRD), thermogravimetric analysis (TGA) and differential scanning calorimetry (DSC) were used to characterize the solidified powder. A portable GC–MS instrument was used to detect volatile components in the air (Fig. 1). The purpose of this project was to explore the volatilization rules of VOs and the deodorization technology of porous starch for VOs to provide a feasible stability technology for VOs.

## Results

### Characterization of porous starch properties

The size of the PS particles was 35.981 μm, the BET specific surface area was 3.9761 m^2^/g, the single-point adsorption of the total pore volume was 0.0053 cm^3^/g, and the average adsorption pore diameter was 5.3304 nm.

### Determination of chemical composition of volatile oil

See Table 1.

**Table 1:** Chemical composition of essential oil.

NO	Chemical composition	Molecular formula
1	beta-Thujene	C_10_H_16_
2	alpha-pinene	C_10_H_16_
3	beta-phellandrene	C_10_H_16_
4	beta-pinene	C_10_H_16_
5	Myrcene	C_10_H_16_
6	alpha-phellandrene	C_10_H_16_
7	alpha-Terpinene	C_10_H_16_
8	Cymene	C_10_H_14_
9	d-limonene	C_10_H_16_
10	beta-Ocimene	C_10_H_16_
11	gamma-Terpinene	C_10_H_16_
12	Terpinolene	C_10_H_16_
13	Linalool	C_10_H_18_O
14	beta- Terpineol	C_10_H_18_O
15	Terpinen-4-ol	C_10_H_18_O
16	alpha-Terpineol	C_10_H_18_O
17	Decanal	C_10_H_20_O
18	(−)-cis-Carveol	C_10_H_16_O
19	Citronellol	C_10_H_20_O
20	Nerol	C_10_H_18_O
21	Thymol methyl ether	C_11_H_16_O
22	d-Carvone	C_10_H_14_O
23	Perillaldehyde	C_10_H_14_O
24	Thymol	C_10_H_14_O
25	2-Methoxy-4-vinylphenol	C_9_H_10_O_2_
26	delta-Elemene	C_15_H_24_
27	beta-Elemene	C_15_H_24_
28	beta-Caryophyllene	C_15_H_24_
29	(−)-gamma-Elemene	C_15_H_24_
30	beta-Farnesene	C_15_H_24_
31	Humulene	C_15_H_24_
32	(−)-Germacrene D	C_15_H_24_
33	alpha-Farnesene	C_15_H_24_
34	(+)-delta-Cadinene	C_15_H_24_

### Volatilization rate of d-limonene in volatile oil

The volatilization rates of d-limonene at 25 °C and 60 °C are calculated. At 25℃, the volatilization rates of d-limonene were 29.28%, 51.15%, 78.63%, 82.89%, 97.31% and 100% respectively at 1 h, 2 h, 4 h, 8 h, 12 h, 24 h. At 2 h, half of the d-limonene had volatilized, and at 12 h, d-limonene had almost completely volatilized. At 60℃, the volatilization rates of d-limonene were 18.58%, 35.90%, 51.47%, 77.25%, and 100% respectively at 0.5 h, 1 h, 1.5 h, 2 h, 3 h. At 60 °C, d-limonene volatilized faster due to the instability of VO at relatively high temperatures, and d-limonene volatilized completely at 3 h.

### Appearance and adsorption rate of porous starch and volatile oil in a solidified cured powder

Different proportions of PS and VO were prepared and cured as a powder. The results showed that when the proportions of VO and PS were 1:5, 2:5, 3:5 and 4:5, the VO could be well absorbed, the dispersion of the powder was relatively uniform and did not stick to the walls or exhibit cohesion phenomena; the mixtures presented a powder state, and the surface of the powder was dry. When the ratio of VO to PS was 5:5, agglomeration occurred, and the prepared powder had an obvious greasy feeling, indicating that the VO proportion had exceeded the maximum adsorption capacity of the PS.

According to the appearance of the solidified PS/VO powder, the adsorption capacity of PS for VO could be preliminarily observed. On this basis, the contents of chemical components in the powders with different proportions of solidified powder were determined, and the adsorption rate of VO in different proportions of solidified powder was calculated. The VO: PS = 1:5, VO: PS = 2:5, VO: PS = 3:5, VO: PS = 4:5, and VO: PS = 5:5 powders adsorption rate is respectively: 44.52%, 57.97%, 51.34%, 50.32%, 41.78%.

### Stability study of PS/VO powders

**Figure 2:**
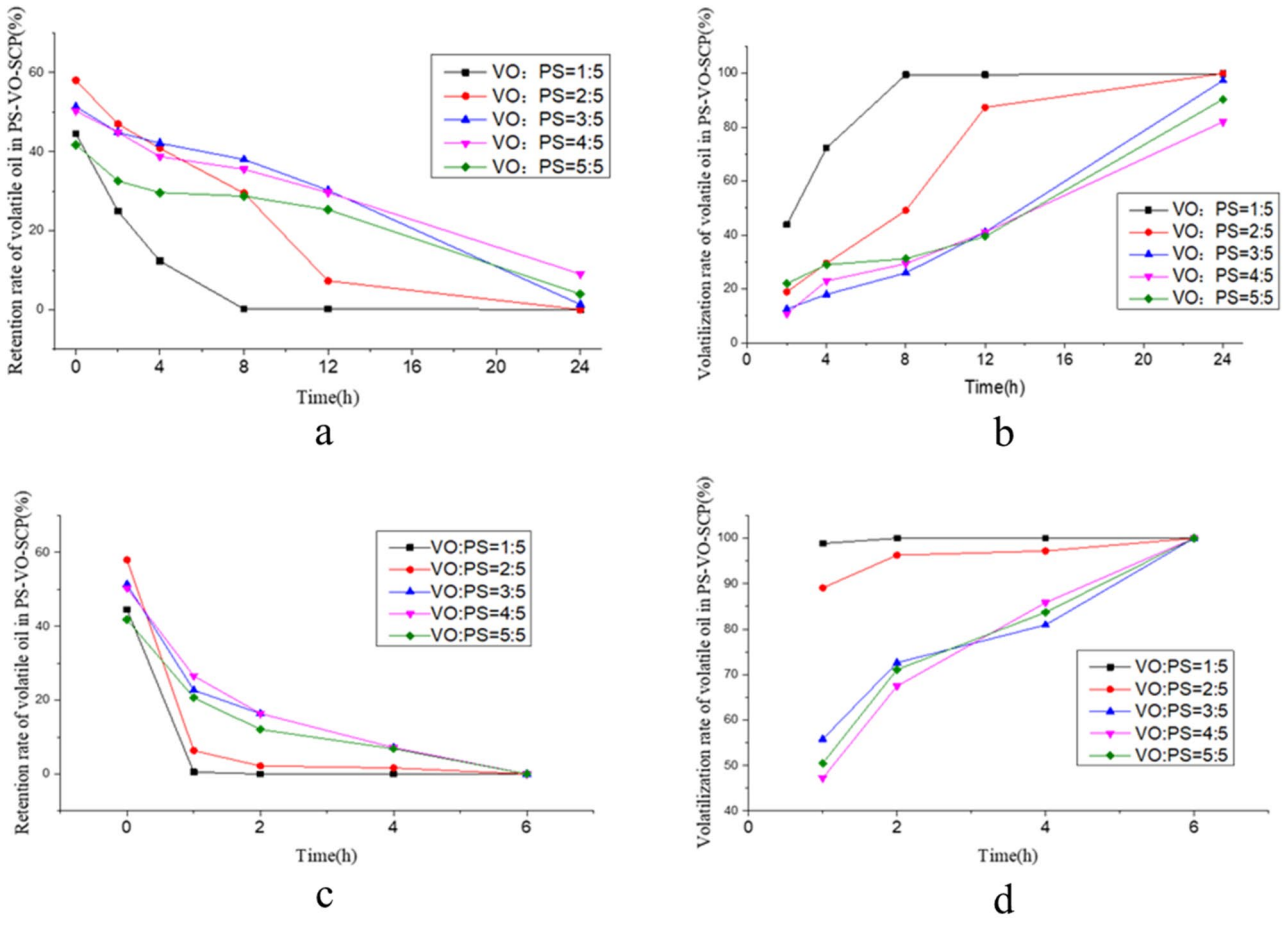
Results of VO/PS powders stability (**a**) Retention curves of volatile oil in PS/VO powders with different proportions at 25 °C. (**b**) Volatilization curves of volatile oil in PS/VO powders at different ratios at 25 °C. (**c**) Retention curves of volatile oil in PS/VO powders with different proportions at 60 °C. (**d**) Volatilization curves of volatile oil in PS/VO powders with different proportions at 60 °C.

VO:PS = 1:5, VO:PS = 2:5, VO:PS = 3:5, VO:PS = 4:5, and VO:PS = 5:5 were prepared with PS and VO. Powders with different VO and PS proportions were stored at 25 °C at 60 °C, sampled at 0 h, 2 h, 4 h, 8 h, 12 h and 24 h. The retention rate of the VO in the different powders was calculated according to the method outline in “Volatilization rules of chemical components in the volatile oil” in section. The retention curve of the VO in the powder versus time was generated. The volatility of VO in the powders with different proportions was calculated, and the curve of volatilization rate of the VO in the powders versus time was plotted (Fig. 2).

According to the stability results of the solidified powder with different proportions of VO and PS at 25 °C and 60 °C, the ratio of VO, the amount of PS, the adsorption rate of VO, the volatilization rate and other factors, the optimum VO:PS ratio was 3:5.

### Stability study of porous starch solidified-volatile oil powder tablets

The VO:PS ratio of 3:5 was the optimal proportion of VO solidified by PS. The powder was pressed by a single stamping machine. The tablets were stored at 25 °C and sampled at different time points, and the retention and volatilization rates were calculated according to the method outlined in “Rules of volatilization of chemical constituents in the PS/VO powders” in section (Table 2). When the solidified powder was prepared into tablets, the retention rate of d-limonene was only 31% initially, i.e., 0 h. The retention rate of d-limonene was 51% in the powder at 0 h. The reason may be that some volatile components were volatilized when the powder was exposed to the air in the process of pressing the tablet, so the initial retention rate of d-limonene in the tablet was low. This low initial volatile oil content increased the volatilization rate.

**Table 2:** Retention and volatilization rates of d-limonene in PS/VO tablets at 25 °C.

Time (h)	Retention rate (%)	Volatilization rate (%)
0	31.03	
2	27.98	9.83
4	22.06	28.90
8	19.56	36.96
12	6.37	79.47
24	0.03	99.91
36	0.02	99.94

### Volatilization rules of chemical components in the volatile oil

**Figure 3:**
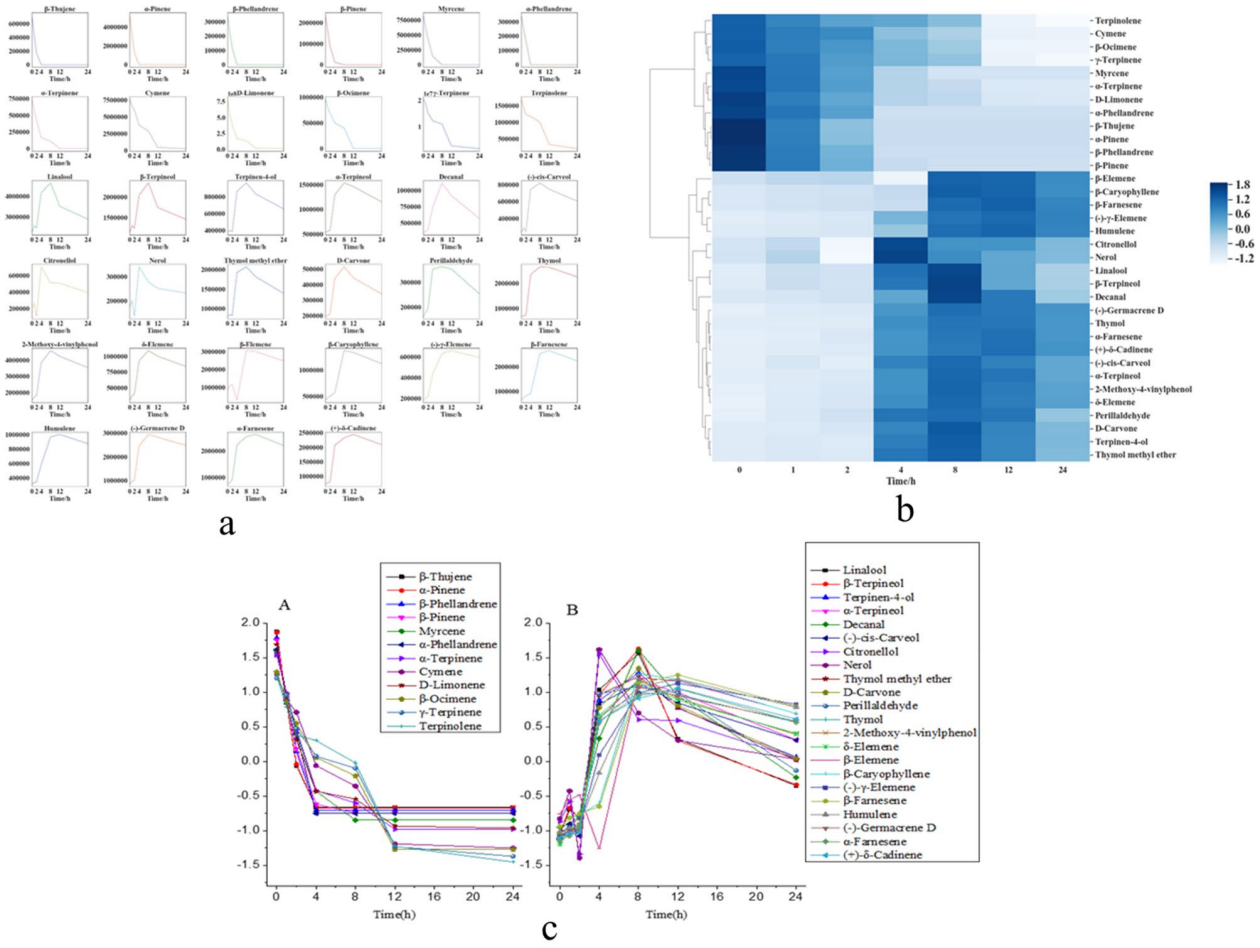
VO statistical analysis. (**a**) Change in the peak areas of chemical constituents in the volatile oil with time. (**b**) Cluster analysis of chemical components in volatile oil (Python v3.7.3 https://www.python.org/). (**c**) Rules of volatilization of class A and B chemical constituents in the volatile oil.

The peak areas of the chemical constituents of the VO measured at 25 °C at different time points were analysed, and the 34 chemical constituents were identified. In the order of retention time, the 34 chemical components were beta-thujene, alpha-pinene, beta-phellandrene, beta-pinene, myrcene, alpha-phellandrene, alpha-terpinene, cymene, d-limonene, beta-ocimene, gamma-terpinene, terpinolene, linalool, beta-terpineol, terpinen-4-ol, alpha-terpineol, decanal, (−)-cis-carveol, citronellol, nerol, thymol methyl ether, D-carvone, perillaldehyde, thymol, 2-methoxy-4-vinylphenol, delta-elemene, beta-elemene, beta-caryophyllene, (−)-gamma-elemene, beta-farnesene, humulene, (−)-germacrene D, alpha-farnesene, and (−)-delta-cadinene. The curve of the peak areas of the 34 chemical components versus time were generated (Fig. 3a). It can be seen from the figure that the changes in peak area with time exhibited certain rules. The curve of chemical components such as beta-thujene, alpha-pinene, beta-pinene, myrcene, d-limonene, and alpha-terpinene showed a consistent change in signal with time. The curves of other chemical components, such as linalool, citronellol, thymol, and alpha-farnesene, were different than those of the above-mentioned components.

Since the peak area values of different chemical components differed greatly, the values were standardized by standard deviation, and then the processed values were analysed by cluster analysis using Python software (Fig. 3b). It can be seen from the figure that the 34 chemical components could be divided into two categories. The 12 compounds terpinolene, cymene, beta-ocimene, gamma-terpinene, myrcene, alpha-terpinene, d-limonene, alpha-phellandrene, beta-thujene, alpha-pinene, beta-phellandrene, and beta-pinene were considered class A chemical components. beta-Elemene, beta-caryophyllene, beta-farnesene, (−)-gamma-elemene, humulene, citronellol, nerol, linalool, beta-terpineol, decanal, (−)-germacrene D, thymol, alpha-farnesene, ( +)-delta-cadinene, (−)-cis-carveol, alpha-terpineol, 2-methoxy-4-vinylphenol, delta-elemene, perillaldehyde, D-carvone, terpinen-4-ol, and thymol methyl ether were the 22 chemical compounds constituting class B.

The peak area values after standardization were plotted over time (Fig. 3c). It can be seen from the figure that the curves of class A compounds were consistent, indicating that class A compounds exhibited a consistent volatilization rule. The curves of the class B components were basically consistent, indicating that the class B components showed a consistent volatilization rule. It can be concluded from the above results that the first 12 eluted compounds were are class A chemical components, all of which are olefins with small molecular weights, high volatilization curve slopes and fast volatilization speeds. The 13th chemical component (linalool), which was an alcohol, began the elution of class B components. The elution of these compounds was later than that of the class A compounds, and the class B components were alcohols, ethers, phenols, aldehydes, etc., with large molecular weights, relatively stable volatilization curve and slow volatilization speeds.

From the above results, it can be concluded that the release rate of essential oil is related to the saturated vapor pressure of the compound, which is mainly affected by molecular weight and material structure. The smaller the molecular weight, the easier it is to escape; The weaker the hydrogen bond and dipole interaction between homologous compounds, the easier it is to escape from gasification. It can be seen from the figure that compounds with small molecular weight and no intramolecular hydrogen bond in volatile oil have fast volatilization rate, such as pinene, limonene, Terpinene, etc. The volatilization rate of compounds with large molecular weight and intramolecular hydrogen bond is slower than the former, such as terpineol, thymol, perilla aldehyde and so on.

### Rules of volatilization of chemical constituents in the PS/VO powders

**Figure 4:**
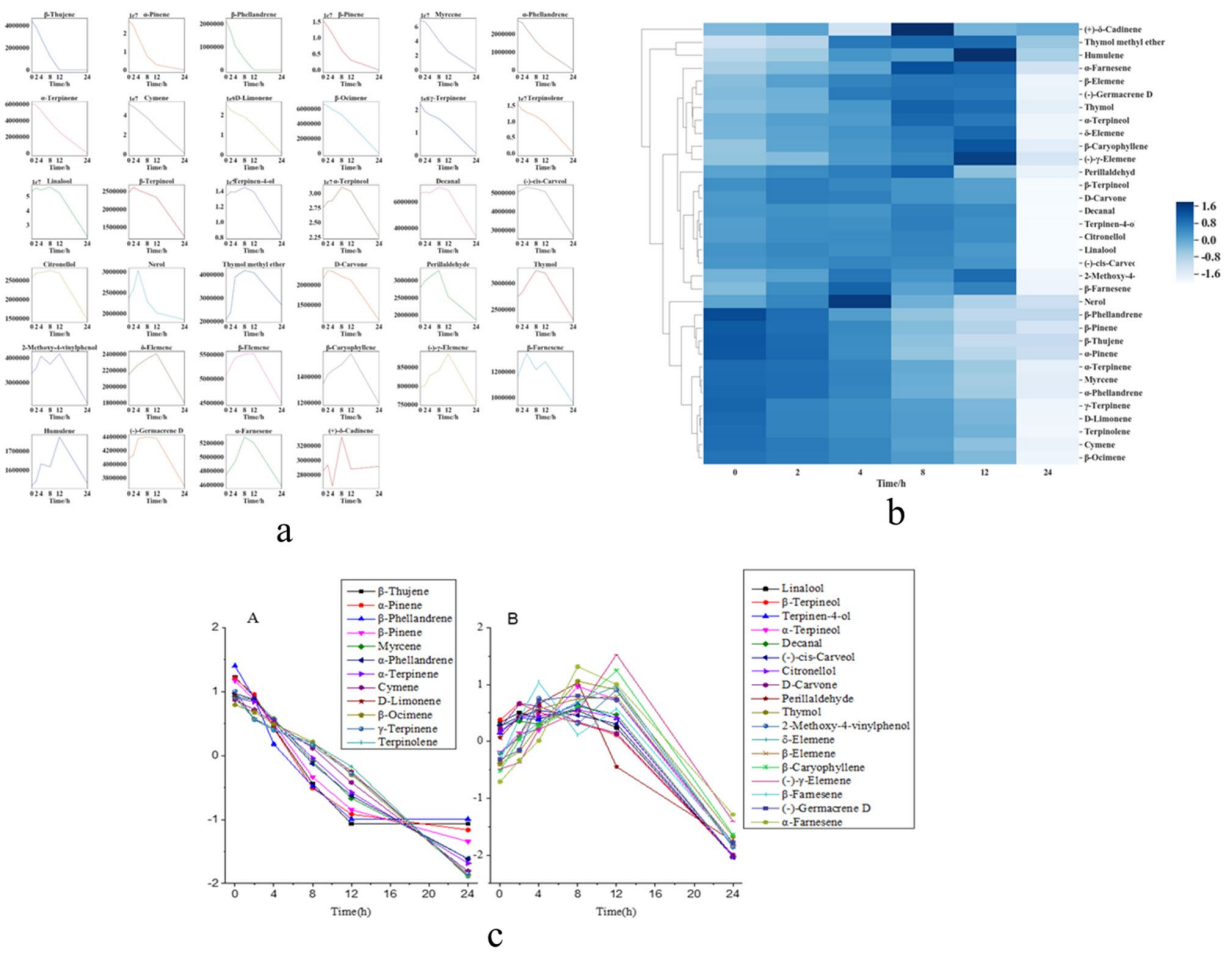
VO/ PS powder statistical analysis. (**a**) The peak areas of volatile oil components in PS/VO powder over time. (**b**) Cluster analysis diagram of the volatile oil chemical components in the PS/VO powder(Python v3.7.3 https://www.python.org/). (**c**) Volatilization rules of volatile oil components in the PS/VO powder.

The VO:PS ratio of 3:5 was the optimal ratio for curing the volatile oil, and the resulting powder was subsequently analysed. The peak areas of the chemical constituents of the VO in the solidified powder were analysed at different time points. As conducted for the chemical constituents of the raw VO, the changes in peak areas over time of the 34 chemical constituents were analysed and plotted, and the analytical method was the same as that in Sect. 3.7.1 (Fig. 4a). Class A chemicals were still grouped together. Most of the chemical components of class B could still be grouped together, but a few of them, such as (+)-delta-cadinene, thymol methyl ether, humulene, and nerol, were relatively dispersed, which might be related to the PS carrier material (Fig. 4b). After Standardization, the peak areas of class A and class B components were plotted versus time (Fig. 4c).

### The volatilization rule of chemical components in the PS/VO tablets

**Figure 5:**
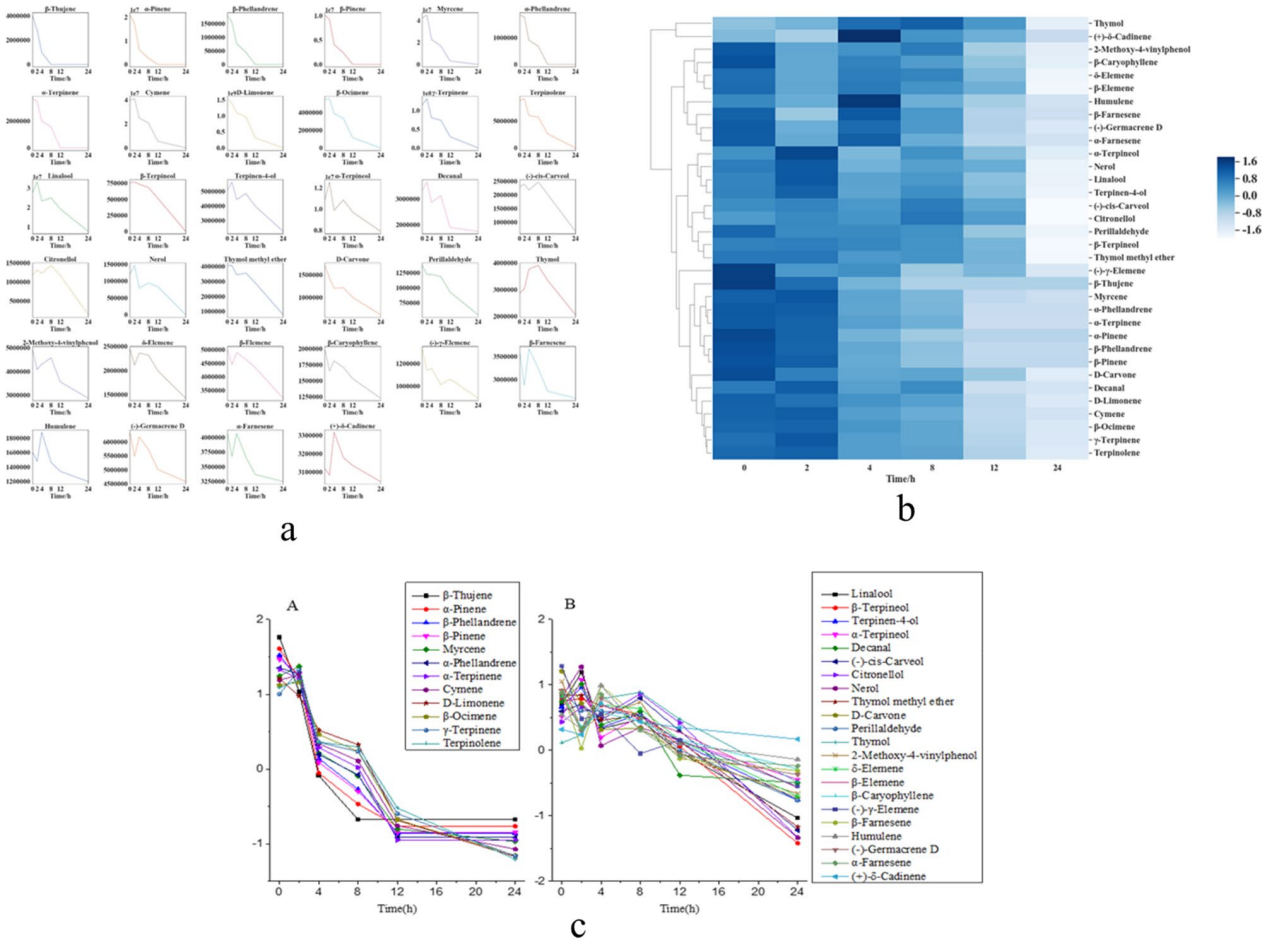
VO/ PS tablets statistical analysis. (**a**) Curve diagram of changes in volatile component peak areas in PS/VO tablets with time. (**b**) Cluster analysis diagram of the volatile oil constituents in the PS/VO tablets (Python v3.7.3 https://www.python.org/). (**c**) The volatilization rules of the class A and B components in PS/VO tablets.

The solidified powder of VO:PS = 3:5 was pressed into tablets. GC–MS was used to detect the chemical composition of the VO in the tablets at different time points. The 34 peak areas of the VO chemical constituents in the tablets at different time points were standardized and analysed to generate the volatilization curves of chemical constituent peak area changes over time (Fig. 5a). The peak area values of the 34 chemical constituents were standardized by standard deviation and analysed by clustering (Fig. 5b). It can be seen from the figure that class A components and class B chemical components were grouped into one class. The normalized peak areas of the class A and class B components were plotted against time (Fig. 5c).

The chemical composition of the VO, cured powder, and tablets changes with time, and clustering analysis showed similarities in all three forms of the VO. The classification of chemical compounds was closely associated with the retention time. The first 12 eluted chemical compounds could be combined into a class (class A), and the 22 other compounds classed them together into class B. Second, the clustering results were closely related to the type of chemical component. Class A chemical components were all olefins, while class B chemical components were alcohols, ethers, phenols, aldehydes, etc. The volatilization rules of class A and class B components were plotted, and it was found that the slope of class A chemical component volatilization was larger than that of class B components, which was consistent with the theory of head, body and base notes in essence and fragrance theory. In the classification of spices, spices were divided into three categories, namely, those having head notes, body notes and base notes, according to the time of volatilization and retention of their fragrance on scented paper.

### Evaluation of the sustained-release effect

**Figure 6:**
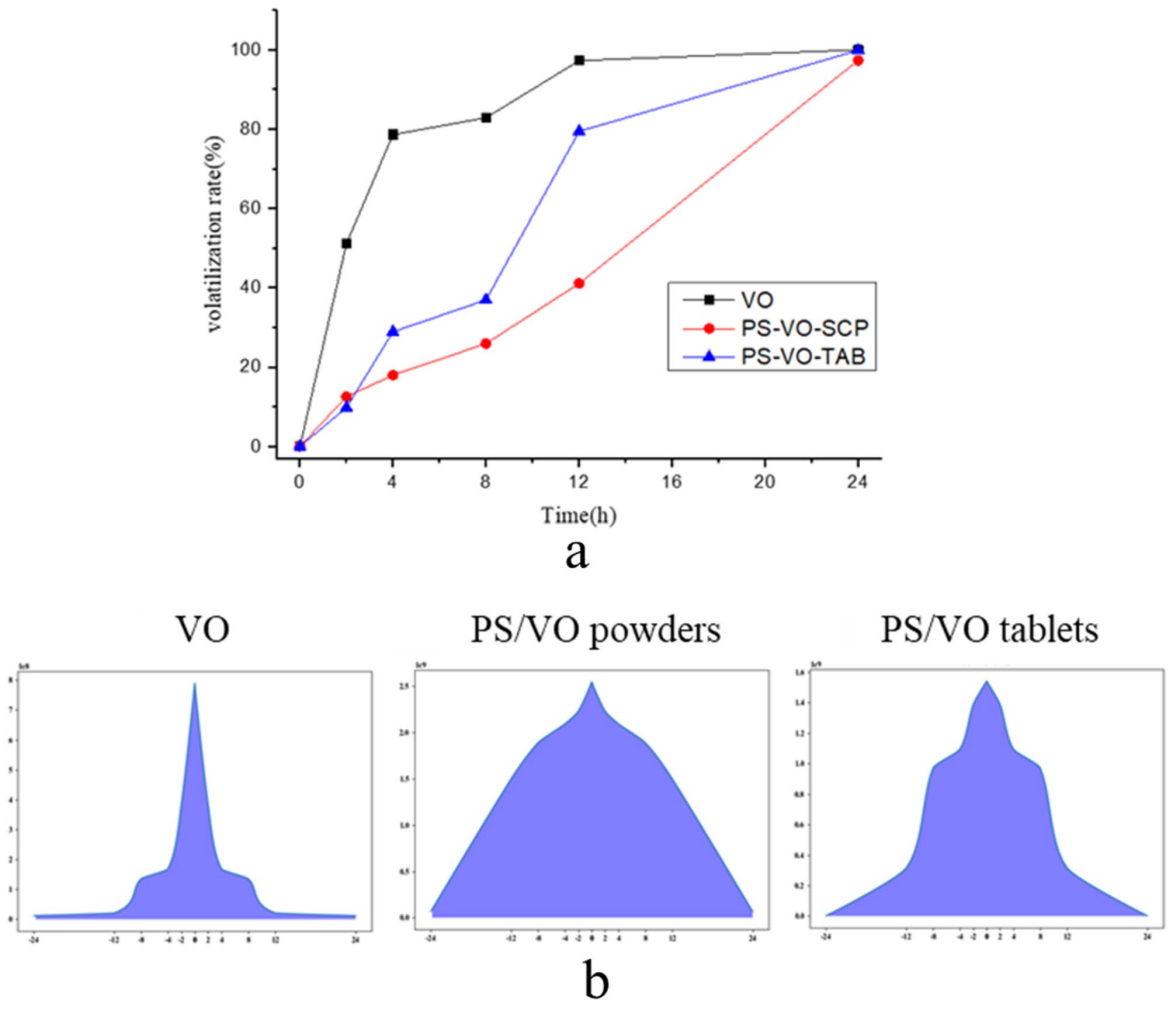
(**a**) d-limonene volatilization curves. (**b**) d-limonene aroma pattern before and after the curing of volatile oil.

The volatilization rate of d-limonene in the VO, PS/VO powder and PS/VO tablets was analysed, and the volatilization curves were generated (Fig. 6a). Because the initial VO content of the tablets was lower than that of powder samples, the volatilization rate of the tablets was faster than that of the powder but still slower than that of the raw VO.

With d-limonene as the index component, the aroma graphs of d-limonene in the VO, PS/VO powder and PS/VO tablets were generated (Fig. 6b). It can be seen from the aroma graph that the VO was volatilized quickly, and the aroma graph shows a sharp peak. After the solidification of the VO in the PS, the release of the VO became slow and stable, achieving the effect of steady and slow release. Compared with that of the PS/VO tablets, the release of VO from the PS/VO powder tended to be more gradual, which was closely related to the retention rate of the initial VO when producing the powder and tablets.

### XRD, TG and DSC characterization of the PS/VO powder

**Figure 7:**
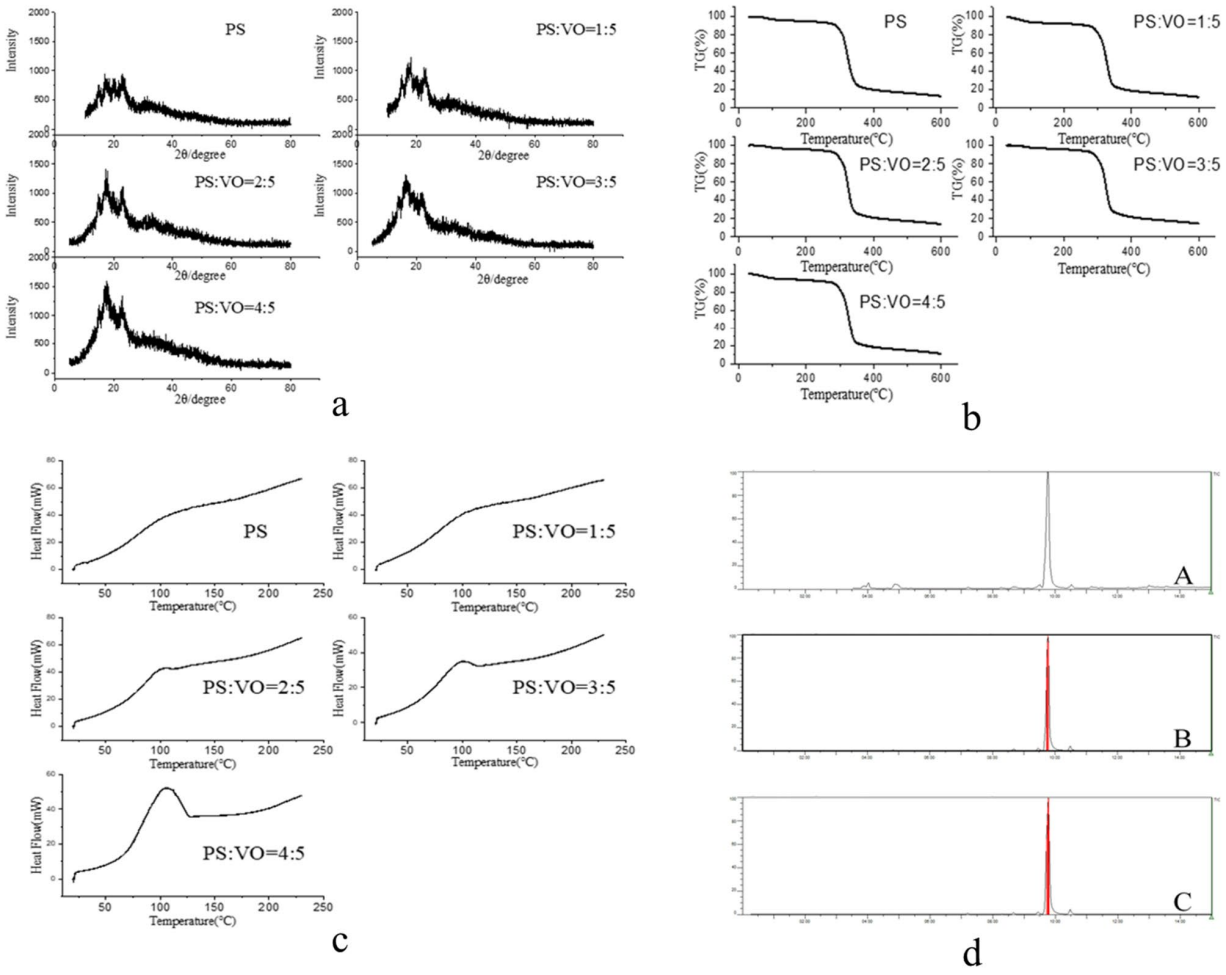
(**a**) X-ray diffraction patterns of the PS/VO powders. (**b**) TG diagram of the PS/VO powders. (**c**) DSC diagram of the PS/VO powders. (**d**) TIC of volatile oil gas composition (A), TIC of gas components in PS/VO powder (B), and TIC of gas components in PS/VO tablets (C).

XRD analysis was performed on PS/VO powder with different proportions (Fig. 7a). Compared with that of PS, the PS/VO powder XRD pattern had a higher characteristic peak, and the larger the proportion of the VO was, the larger the characteristic peak, indicating that the PS absorbed the VO, and the powder surface also contained a certain amount of volatile oil, so the PS/VO powder X-ray diffraction pattern had a peak more characteristic peak of VO than of PS^18,19^.

Thermal analysis can characterize the changes in physical and chemical properties of a material, and TGA was performed on PS/VO powders with different VO and PS proportions (Fig. 7b). It can be seen from the figure that the thermogravimetric diagram of PS before and after curing with VO was consistent, indicating that the PS had no effect on the physical and chemical properties of the VO^20^.

DSC can reflect the combined characteristics of a drug and carrier, and PS/VO powders with different PS and VO proportions were analysed via DSC (Fig. 7c). It can be seen from the figure that PS/VO powders with different VO and PS proportions all exhibited exothermic peaks at 104 °C. The larger the proportion of the VO was, the larger the exothermic peak. The above results were consistent, indicating that the PS did not change substantially after curing the VO, indicating that the mechanism by which the PS and VO were combined was physical adsorption^21^.

### VOC analysis before and after curing of volatile oil

The VOCs of the VO, PS/VO powder and PS/VO tablets in the air were measured by portable GC–MS, and according to the NIST database, the chemical components of the VO, PS/VO powder and PS/VO tablets were detected to be alpha-pinene, beta-pinene, alpha-terpinene, cymene, d-limonene, and terpinolene. In the three forms of raw VO, PS/VO powder and PS/VO tablets, the chemical constituents of the VO that volatilized into the air were consistent, indicating that there was no chemical change after VO curing and after tablet pressing, and the adsorption mechanism of the VO by PS was physical.

d-limonene accounted for more than 80% of the VO composition in dried tangerine peel and immature tangerine peel and was therefore easily detected. However, as the instrument sensitivity of the portable GC–MS is in the range of low-to-medium ppb, when other chemical components at low levels were detected, d-limonene exceeded the maximum value of detection by the instrument, and the peak area appeared as a red warning (Fig. 7d). The red area is the peak area of d-limonene. Therefore, this instrument could only detect high contents of alpha-pinene, beta-pinene, alpha-terpinene, cymene, d-limonene, terpinolene, etc., while other volatile components could not be detected.

## Conclusion

Volatilization rules of the volatile oil are related to chemical composition and aroma theory. Based on the study of volatilization rules, the chemical constituents of the VO were grouped into two categories according to their retention times. First, the classification of compounds into these two classes was closely related to the retention time, and their classification accuracy was verified statistically. Second, the compound classification was closely related to the type of chemical component, and this was also the first study to innovatively associate volatilization rules with chemical compound type. At the same time, the volatilization rules were consistent with the aroma theory of head notes, body notes and base notes. The volatile oil cured by porous starch had optimized volatilization and stable release. The study on the stability of the solidified VO powder produced with PS and the evaluation of the slow-release effect showed that the release of the VO had changed to some extent after processing, and the release of the VO could be regulated by the PS to achieve optimal release and stability. The XRD, TGA and DSC characterizations of the VO solidified by PS showed that the physical properties of the VO did not change after solidification. The VOCs of VO before and after curing were analysed for the first time in this paper. The results showed that the volatile chemical composition of the VO did not change after curing, indicating that the adsorption of the VO by PS was a form of physical adsorption.

The volatile oil of traditional Chinese medicine is highly volatile, the effective components are easy to be lost, and it is unstable to air, light and heat, which makes it difficult for the stable release and administration of traditional Chinese medicine volatile oil, affects the clinical efficacy of volatile oil preparation, and has become an important bottleneck in the development of traditional Chinese medicine volatile oil. Focusing on the key problems perplexing the volatile oil of traditional Chinese medicine (strong volatility and poor stability), “adsorption–desorption” curing technology enables porous carrier materials to regulate the stable release of traditional Chinese medicine volatile oil, improve the volatility and stability of volatile oil, improve the quality of traditional Chinese medicine volatile oil preparation, realize stable release and administration, and ensure the effectiveness. “Adsorption–desorption” curing technology not only solves the problem of traditional Chinese medicine volatile oil, but also innovates the key technology of traditional Chinese medicine volatile oil preparation and endows the volatile oil with modern scientific and technological connotation.

## Materials and methods

### Instruments and materials

The following instruments were used in this study: an RW50 top electric mixer (Shanghai Analysis), a YP2002 electronic balance (Shanghai Yongzheng Medical Instrument Co., LTD.), an Agilent 7890A/5975C gas chromatography mass spectrometer (Agilent, USA), a D8 ADVANCE X-ray diffractometer (BRUKER, Germany), a TGA4000 thermogravimetric analyser (PE), a DIAMOND DSC (US PE Company), an MS2000 laser particle size analyser (Malvern), Tristar II Plus 2.02 BET physical sorbent (MAC, USA), a HAPSITE ER Portable GC–MS (INFICON inc., USA), a Bsa224s-cw electronic balance (Satris Scientific Instrument (Beijing) Co., LTD.), and a DP30A single stamping machine (Beijing National Medicine Longli Technology Co., LTD.). Anhydrous ethanol (batch Number: 190905, Xilong Scientific Co., Ltd.) and porous starch (batch no. 20190325, Liaoning Lida Biotechnology Co., LTD.) were also used in this study.

### Determination of the physical properties of porous starch

The particle size of porous starch *(*PS) was measured using a Malvern laser particle size meter; air was the dispersion medium, the feed vibration was 70%, and the pressure of the dispersed starch was 2.00 Bar. An appropriate amount of PS was placed in a sample tube and dried to constant weight with nitrogen at 60 °C. The surface area, porosity and pore size of PS were measured by a specific surface area meter.

### Determination of the volatile oil chemical composition

VO (100 μL) was placed in a 10 mL brown bottle, stabilized with anhydrous ethanol, shaken well, and filtered with a 0.22 μm microporous membrane filter. The filtrate was then analysed by GC–MS. The gas chromatographic conditions were as follows: 13,105 Chiral Cap. Column Rt-bDEXsm (30 m × 250 μm × 0.25 μm); carrier gas, helium; flow rate, 1.0 mL/min; sample volume, 1.0 µL; split ratio, 40:1; inlet temperature, 250 °C; and temperature programme, 40 °C, holding for 2 min, 10 °C/min to 220 °C, 20 °C/min to 300 °C, holding for 5 min. The mass spectrometric conditions were as follows: ionization source, EI; electron energy, 70 eV; ionization source temperature, 230 °C; quadrupole temperature: 150 °C; and scan mode: full scan^22,23^.

### Volatilization rate of d-limonene in volatile oil

An appropriate amount of VO was placed in a surface dish and sampled at different time points at 25 °C and 60 °C. The chemical composition of the VO was detected by GC–MS. Using d-limonene as the index component and its peak area in the initial VO as the standard, the volatilization rate of d-limonene in the VO at different time points was calculated (Formula 1).

1 $${\text{Volatilization}}\;{\text{rate}}\;{\text{of}}\;{\text{volatile}}\;{\text{oil}} = \left( {{\text{initial}}\;{\text{peak}}\;{\text{area}} - {\text{peak}}\;{\text{area}}} \right)/{\text{initial}}\;{\text{peak}}\;{\text{area}} \times 100\%$$

### Preparation of volatile oil solidified by porous starch

A predetermined amount of PS was placed in a container according to the VO (mL):PS (g) ratios of 1:5, 2:5, 3:5, 4:5 and 5:5. The PS and VO mixture was stirred at 200 r/min for 8 min for the volatile oil to be fully absorbed by the porous starch. The VO powder was cured, sealed for preservation, and stored until use.

### Adsorption rate of the volatile oil solidified by porous starch

VO solidified by PS (1.5 g) was weighed accurately and put into a conical flask. Then, 25 mL of absolute ethanol was added. The mixture was weighed, ultrasonicated for 10 min, cooled, weighed again, brought to the initial weight with anhydrous ethanol, shaken well, and filtered. The mixture was filtered through a 0.22 μm microporous membrane, and the chemical composition of the filtrate was determined by GC–MS. Taking the peak area of d-limonene in the VO as the standard peak area, the adsorption rate of the VO by PS at different proportions was calculated (Formula 2).

2 $$\begin{aligned} {\text{Adsorption}}\;{\text{rate}}\;{\text{of}}\;{\text{volatile}}\;{\text{oil}}\;{\text{in}}\;{\text{solidified}}\;{\text{powder}} = & {\text{actual}}\;{\text{peak}}\;{\text{area}}\;{\text{of}}\;{\text{D}} \\ & \quad - {\text{limonene}}\;{\text{in}}\;{\text{solidified}}\;{\text{powder}}/{\text{theoretical}}\;{\text{peak}}\;{\text{area}}\;{\text{of}}\;{\text{D}} \\ & & \quad - {\text{limonene}}\;{\text{in}}\;{\text{solidified}}\;{\text{powder}} \times 100\% \\ \end{aligned}$$

### Stability of the volatile oil solidified by porous starch

Solidified VO powders with different proportions of PS and VO were prepared. The solidified VO powder was spread in a container at a thickness of approximately 0.5 cm. The solidified powder was placed at 25 °C and 60 °C. Samples were taken at different time points. The samples were prepared according to the method outlined in “Stability study of porous starch solidified-volatile oil powder tablets” section, and the chemical composition was determined. Taking d-limonene as the index compound and the peak area of d-limonene in the added VO as the standard, the retention rate (Formula 3) and volatility rate (formula 4) of the VO in the solidified powder at different time points were calculated^24^.

3 $${\text{The}}\;{\text{retention}}\;{\text{rate}} = {\text{actual}}\;{\text{peak}}\;{\text{area}}\;{\text{of}}\;{\text{D}} - {\text{limonene}}\;{\text{in}}\;{\text{solidified}}\;{\text{powder}}/{\text{theoretical}}\;{\text{peak}}\;{\text{area}}\;{\text{of}}\;{\text{D}} - {\text{limonene}}\;{\text{in}}\;{\text{solidified}}\;{\text{powder}} \times 100\%$$

4 $${\text{The}}\;{\text{volatility}}\;{\text{rate}} = \left( {{\text{initial}}\;{\text{retention}}\;{\text{rate}} - {\text{retention}}\;{\text{rate}}} \right)/{\text{initial}}\;{\text{retention}}\;{\text{rate}} \times 100\%$$

### Stability of cured tablets

The cured powder with the optimal proportions the VO and PS was pressed, and the sheet weight and pressure of the single stamping machine were adjusted so that the cured powder could be pressed into tablets that met the standard requirements of medicinal tablets. The effect of the tablet form on the volatilization rate was investigated. The solidified tablets were placed at 25 °C and sampled at different time points. Four solidified tablets were weighed accurately placed in a conical flask, combined with 25 mL of anhydrous alcohol, weighed again, ultrasonicated for 10 min, cooled, weighed, and brought up to the initial weight with anhydrous alcohol. The mixture was shaken well, filtered with a 0.22 μm microporous membrane filter, and analysed by GC–MS. The retention rate (formula 3) and volatilization rate (formula 4) of the VO in the cured tablets at different time points were calculated.

### Investigation of volatilization rules of the volatile oil before and after curing

The peak area of the chemical constituents of the VO changed with time, and a total of 34 chemical constituents were detected. Python software was used to generate the release curves of the 34 chemical constituents in the VO over time according to changes in their peak areas. Python software was used to standardize the standard deviations of the peak areas of all chemical components and clustered the volatilization rules of the 34 chemical components. According to the clustering results, the 34 chemical components were classified into two categories, and the curve of the peak values after standardization of these two categories versus time was plotted. The peak area of 34 chemical components in the PS cured powder and the PS cured tablets changed with time, and the analytical method of the tablets was consistent with that of the powder. The release of the VO in the solidified powder and the solidified tablet and the influence of PS on the release of the VO were investigated^25^.

### Evaluation of the sustained-release effect of volatile oil before and after curing

With d-limonene as the index compound, the volatilization rate curves of the VO, solidified powder and solidified tablets versus time were plotted to evaluate the sustained-release effect. At the same time, the curve of the peak area of the d-limonene chemical composition in the VO, cured powder and cured tablet changed with time, and the axial symmetry image was included with the aroma graph of d-limonene. The change in the aroma graph before and after curing of the VO was compared to evaluate its slow-release effect.

### XRD, TG and DSC characterization of volatile oil solidified by porous starch

XRD was performed on a solidified PS/VO powder sample at temperatures from 10 to 80 °C. TGA was performed on the solidified powder with a heating rate of 10 °C·min^−1^ from 30 °C to 600 °C in nitrogen, and DSC was performed on the solidified powder with a heating rate of 10 °C·min^−1^ from 30 °C to 450 °C in nitrogen.

### VOC analysis before and after curing of volatile oil

Portable GC–MS instruments are sensitive in the low-to-medium ppb range for the analysis of volatile organic compounds (VOCs) in the air. According to the characteristics of the instrument, the VO, solidified powder and solidified tablet samples were placed in a sealed collector and volatilized naturally, and the volatile components of the gas in the collector were determined by portable GC–MS. The detection mass range was m/z 45–250, the air intake was 40 mL, the filament delay time was 210 s, the inlet line cleaning time was 1 min, and the sample collection time was 1 min. The chemical constituents of the VO released into the air before and after curing were detected, and the changes in volatile components before and after curing were evaluated.
